# Cases of Tic Douloureux; in One of Which the Patient Took about Eight Ounces of Carbonate of Iron in Thirty-Six Hours

**Published:** 1826-02

**Authors:** P. C. Blackett


Art. VIII.—*Cases of Tic Doulourex; in one of which the Patient took about eight Ounces of Carbonate of Iron in thirty-six Hours.* Communicated by P. C. Blackett, Esq. &c.

CASE I.

Mrs. C—, a widow, ætat. forty-three, of temperate habits and general good health, (except occasional colds, with rheumatism, &c. &c.) in January last applied to me, and complained of an occasional pain of the right side of her face, which had tormented her for upwards of two years, immediately in the vicinity of the parotid gland, and behind the ear of the same side: in the day-time she would have three or four paroxysms, but at night, or whilst in her bed, she was totally free from them. I found, on examination, the portio dura of the auditory nerve affected, very sensible, and bringing on an immediate paroxysm by the slightest touch. It was evident that the affection was in that part of the nerve, after it had given off the superior and inferior ramus, where it advances forward and through the parotid gland, to which it gives several filaments; some of these slender ramifications running from without internally, and surrounding that branch of the external carotid artery which goes behind the ear. Regarding her complaint as produced by some irritation of the womb, caused by that period in the constitution of women commonly called *the change of life*, I gave her, after having regulated the bowels, the carbonate of iron, according to the plan of Mr. Hutchinson. She continued the remedy for nine or ten days, when the intensity of the pain was somewhat alleviated, and the frequency of the paroxysms reduced to only one in the twenty-four hours.

I advised her to persist in the use of the carbonate of iron for another week; but, at the end of that time, she continued the same as when I last saw her. I then recommended a blister to be applied behind the ear of the affected side, and to be kept open. This plan, with the use of the carbonate, was continued for eight days more: still she continued as before. I then omitted the remedies altogether, and gave her twenty drops of the tincture of belladonna three times a-day. This gradually, in the course of five days, relieved her completely of the paroxysm and the pain. Fearful of a relapse, I ordered her to continue the dose twice a-day for a fortnight; and she has not, since the first remission, complained of the disease.

CASE II.

Captain S—, ætat. twenty-six, in January 1823, complained of great irritation in the urethra and the bladder, with a constant desire to make water, especially after dinner: the urine flowed sometimes in a full stream, and at others in very small quantities. These symptoms of irritability of the bladder and the urethra were always attended with pain over the left eye. I pressed the first branch of the ophthalmic, or first branch of the fifth pair, and found that it gave him great pain; which, however, soon subsided. His description of the pain was, in every sense of the word, tic douloureux. I passed a full-sized bougie into the bladder; which operation also caused a pain in the nerve of the forehead. Considering that some tonic, as well as sedative, was necessary for his complaint, I ordered him the following pills; and to take castor-oil whenever his bowels were constipated.

℞. Cupri Sulphatis, gr. iij.

Extracti Conii, ʒj. M. fiat massa in pilulas xxiv. quarum capiat unam 6ta quâque horâ.

In a week he was much relieved of the irritation of the bladder and the urethra, and in six weeks of the pain in the forehead. His appetite returned; he slept well, and continued to void his urine with ease, when I left him; which was three months after first attending him.

CASE III.

*Letter from a Gentleman at Welford to* Mr. Blackett, *detailing his Case.*

*15th November*, 1825.

In reading the Medical and Physical Journal for October, page 288, I find a Treatise on the Tic Douloureux, and the mode of Treatment, written by you. I was pleased with your description, and particularly so because I have been labouring under the same tormenting malady for this last year, at intervals: but, at the same time, I was disappointed in not finding you so warm in your recommendation of a curative remedy, as in the description of the symptoms and nature of the disease. I am happy to say that, according to the plan of Mr. Hutchinson, the disease was arrested in about thirty-six hours; and I hope and trust that it may hold good, and that I may not have a relapse.

This time twelvemonths I was riding on horseback without a great coat, and without the usual precaution of tying a silk handkerchief round my neck; the wind blowing north-east, and a damp atmosphere. I felt an unpleasant cold sensation upon the zygomatic arch: this daily increased, until I experienced at times great pain, yet such as could be tolerably borne. Since that time I have had a few attacks more severe, and which induced me to try one-drachm doses of the carbonate of iron every six hours, mixed with honey; and a draught with it of the decoction of bark and infusion of cascarilla, with sulphuric acid. This gave relief, but did not cure the disease.

The next attack, I tried gum assafœtidæ, ferri sulphatis āā ʒss. fiat massa, et divide in pilulas xij. I took nine of these pills in the evening of the attack; and the draught, with bark, as before. This gave me more relief than the other, but appeared not sufficient to arrest the disease.

The next attack was in the evening of Sunday, after being at church. I felt a stream of cold air upon the vulnerable part, and expected another touch of it when I got home. In the evening I was seized with a paroxysm, more violent than any before. I directed my assistant to bring me a large tea-cupful of the carbonate of iron, mixed with honey. I immediately took four large tea-spoonsful of it, with a draught, containing one drachm and a half of the extract of sarsæ, and an ounce and a half of peppermint water, made doubly strong with the essential oil. I went to bed, and perspired through the night. My pain became tolerable. *I persevered in the same doses every six hours, day and night, so that the tea-cup, which contained eight ounces of the carbonate of iron, I took at six times:* at that rate, the quantity I took for each dose was one ounce and two drachms, accompanied with one drachm and a half of the extract of sarsæ, every six hours. This plan was continued for two nights and one day; the disease being arrested. I then took the same quantity of iron twice a-day, and the dose of sarsaparilla at night, for a few days; and I am in great hopes of not having a return.

It proves what large doses of medicine can be borne, when under the influence of great excitement. The medicine had the effect with me of opening the bowels plentifully, but without griping.

I also applied a cataplasm, made with two table-spoonsful of oatmeal, one table-spoonful of flour of mustard, and white wine vinegar heated, to make it into a proper consistence: this I kept to the part for a few minutes at a time, as long as I could bear the heat.

11, *Park-street, Grosvenor-square;*
*8th December,* 1825.

[By another letter, dated December 22d, it appears that this gentleman continued quite well. We have not deemed it necessary to insert either of the rejoinders.—EDITORS.]

